# miR‐422a suppresses SMAD4 protein expression and promotes resistance to muscle loss

**DOI:** 10.1002/jcsm.12236

**Published:** 2017-10-06

**Authors:** Richard Paul, Jen Lee, Anna V. Donaldson, Martin Connolly, Mohammad Sharif, Samantha Amanda Natanek, Ulrich Rosendahl, Michael I. Polkey, Mark Griffiths, Paul R. Kemp

**Affiliations:** ^1^ Molecular Medicine Section, National Heart & Lung Institute Imperial College London South Kensington Campus London SW7 2AZ UK; ^2^ National Institute for Health Research Respiratory Biomedical Research Unit at Royal Brompton and Harefield NHS Foundation Trust and Imperial College London SW3 6NP UK; ^3^ Department of Cardiothoracic Surgery Royal Brompton and Harefield NHS Foundation Trust London SW3 6NP UK; ^4^ Inflammation, Regeneration and Development, National Heart and Lung Institute Imperial College London South Kensington Campus London SW7 2AZ UK

**Keywords:** MicroRNA, TGF‐beta signalling, Muscle wasting, Susceptibility

## Abstract

**Background:**

Loss of muscle mass and strength are important sequelae of chronic disease, but the response of individuals is remarkably variable, suggesting important genetic and epigenetic modulators of muscle homeostasis. Such factors are likely to modify the activity of pathways that regulate wasting, but to date, few such factors have been identified.

**Methods:**

The effect of miR‐422a on SMAD4 expression and transforming growth factor (TGF)‐β signalling were determined by western blotting and luciferase assay. miRNA expression was determined by qPCR in plasma and muscle biopsy samples from a cross‐sectional study of patients with chronic obstructive pulmonary disease (COPD) and a longitudinal study of patients undergoing aortic surgery, who were subsequently admitted to the intensive care unit (ICU).

**Results:**

miR‐422a was identified, by a screen, as a microRNA that was present in the plasma of patients with COPD and negatively associated with muscle strength as well as being readily detectable in the muscle of patients. *In vitro*, miR‐422a suppressed SMAD4 expression and inhibited TGF‐beta and bone morphogenetic protein‐dependent luciferase activity in muscle cells. In male patients with COPD and those undergoing aortic surgery and on the ICU, a model of ICU‐associated muscle weakness, quadriceps expression of miR‐422a was positively associated with muscle strength (maximal voluntary contraction r = 0.59, P < 0.001 and r = 0.51, P = 0.004, for COPD and aortic surgery, respectively). Furthermore, pre‐surgery levels of miR‐422a were inversely associated with the amount of muscle that would be lost in the first post‐operative week (r = −0.57, P < 0.001).

**Conclusions:**

These data suggest that differences in miR‐422a expression contribute to the susceptibility to muscle wasting associated with chronic and acute disease and that at least part of this activity may be mediated by reduced TGF‐beta signalling in skeletal muscle.

## Introduction

Loss of muscle mass and strength are common complications of a range of chronic diseases, including chronic obstructive pulmonary disease (COPD)^1^ and heart failure.^2^ Similarly, muscle mass can be markedly reduced following admission to an intensive care unit (ICU), either in response to surgery^3^ or to critical illness.^4^ In all of these conditions, muscle wasting increases dependency and reliance on social care and is associated with increased mortality.^5^

The prevalence of muscle weakness has been reported to be approximately 35% in COPD^6^ and 20% in heart failure.^7^ Although the presence of chronic disease is a factor driving muscle wasting, there is a poor correlation between classical measures of disease severity (e.g. the Global Initiative for Chronic Obstructive Lung Disease classification of COPD^8^ or New York Heart Association classification of cardiac disease^9^) and muscle wasting. This disparity suggests that some individuals are more susceptible to wasting than others. The variation in response may result from differences in the strength of the atrophic signal sent to the muscle for a given disease severity, the sensitivity of the individual to the atrophic signal or other factors affecting muscle homeostasis including relative rates of muscle regeneration, each of which will be affected by a mixture of environmental (e.g. pre‐existing levels of physical activity and smoking), genetic^10^, ^11^ and epigenetic factors. We have shown that expression of microRNAs (miRNAs) from imprinted loci (C19MC miRNAs and miR‐675) is associated with fat free mass index (FFMI), a marker of muscle bulk in patients with COPD^12^ with miRNAs from the C19MC positively associated with FFMI in men and miR‐657 negatively associated with FFMI in all patients. These epigenetic factors may contribute to the relative rate of regeneration, an observation supported by the reduced presence of centralized nuclei in the muscle of cachectic patients with COPD compared with those with a normal FFMI.^12^, ^13^ The relative importance of these factors will depend on the nature and strength of the atrophic signal.

Ligands from the transforming growth factor beta (TGF‐β) pathway are important regulators of muscle mass that are positively associated with muscle wasting in a variety of conditions. Both myostatin and growth differentiation factor 15 (GDF‐15) are elevated in a range of muscle wasting conditions and are able to promote atrophy.^14^, ^15^, ^16^ Myostatin promotes muscle wasting by activating the canonical TGF‐β signalling pathway via phosphorylation of SMAD proteins, leading to increased protein breakdown and autophagy.^17^, ^18^ SMAD2/3 can also be activated in the absence of exogenous ligand by miR‐542‐5p, a miRNA quadriceps expression of which is positively associated with lung disease severity in patients with COPD.^19^

The importance of the canonical TGF‐β signalling system in the control of muscle mass implies that miRNAs that alter the expression of components of the system will modify the sensitivity of individuals to atrophic signalling. For example, miR‐1 and miR‐181 suppress TGF‐β signalling either indirectly through the suppression of HDAC4 expression and the associated increase in follistatin expression^20^ in the case of miR‐1 or directly in the case of miR‐181.^15^ As both miRNAs are suppressed by GDF‐15 in muscle, one effect of GDF‐15 will be to sensitize cells to TGF‐β and myostatin.^15^

Having established a miRNA pattern in the quadriceps associated with a low FFMI in patients with COPD, we hypothesized that patients susceptible to muscle atrophy would have a different plasma miRNA profile from those who retained muscle. We therefore screened miRNAs in plasma from patients with a low or a retained FFMI. One of the miRNAs that we identified by this screen (miR‐422a) was also readily detectable in muscle. Bio‐informatic analysis using miRWalk^21^ suggested that miR‐422a would target SMAD4, a central component of the canonical TGF‐β signalling pathway. We therefore determined its effect on SMAD4 expression and on TGF‐β signalling in muscle cells. We then analysed its expression in muscle and its association with muscle mass and strength in patients with COPD and in patients requiring aortic surgery, a model of ICU‐associated muscle weakness that we have recently characterized.^3^

## Methods

### Patient cohorts

#### Chronic obstructive pulmonary disease cohort

The cohort of patients with COPD has been described previously.^22^ Briefly, patients were recruited from clinics at the Royal Brompton Hospital. Exclusion criteria included renal, liver or heart failure or a moderate/severe exacerbation in the preceding 4 weeks. Healthy age‐matched controls were recruited by advertisement. Written informed consent was obtained from all patients, and the protocol was approved by the appropriate research ethics committee (studies 06/Q0404/35 and 06/Q0410/54). Demographic data for the patients used in the screen are given in Table S1 and those in the validation study in *Table* 1. Fat free mass was determined by bioelectrical impedance (Bodystat 1500, Bodystat, UK) in patients who had been resting supine for 10 min. Quadriceps strength was measured as supine maximal voluntary contraction (MVC) as described previously and physical performance as 6 min walk distance according to ATS 2002 guidelines.^23^ A biopsy of vastus lateralis in the leg tested for strength was performed under local anaesthetic by the Bergstrom technique.^24^

**Table 1:** Demographic characteristics of the patients with COPD

	COPD (*n* = 52)	Control (*n* = 16)	*P* value
Age	66 ± 8	65 ± 8	NS
Male/female	29 M, 23 F	6 M, 10 F	
Smoking history (pack year)	42 (27.5, 60)	0 (0, 10)	<0.001
Weight (kg)	65.7 (58.4, 76.1)	65.4 (61.0, 74.1)	NS
BMI (kg/m^2^)	23.3 (21.4, 26.3)	24.8 (23.5, 26.2)	NS
FFMI (kg/m^2^)	15.3 (14.5, 16.8)	16.0 (16.9, 15.2)	NS
FEV_1_%	41.1 (27.0, 48.5)	106.8 (100.5, 111.5)	<0.001
TLCO%	45.0 (32.1, 52.4)	87.5 (80.8, 98.1)	<0.001
6MWD%	81 (58, 91)	129 (123, 130)	<0.001
MVC (kg)	26.7 (20.6, 34)	34.1 (27.9, 37.2)	0.032
MVC/FFM (kg)	0.64 (0.51, 0.75)	0.73 (0.61, 0.87)	0.045

Data are presented as mean ± SD for normally distributed data and as median (interquartile range) for data that is not normally distributed.

BMI, body mass index; FFMI, fat free mass index; FEV_1_%, forced expiratory volume in 1 s (% of predicted value); TLCO, transfer capacity of the lung for CO (% of predicted value); 6MWD%, 6 min walk distance (% of predicted value); MVC, maximum voluntary contraction.

#### Aortic surgery cohort

Patients undergoing elective aortic surgery at the Royal Brompton Hospital were recruited to the study and provided written informed consent. The study was approved by the National Research Ethics Committee (07/Q0204/68). The principal inclusion criterion was an elective aortic operation requiring admission to the ICU as identified by the surgical team. Exclusion criteria included pre‐existing muscular or neuromuscular disease, malignancy or contra‐indication to muscle biopsy. Rectus femoris cross‐sectional area (RF_CSA_) was determined by ultrasound^25^ before and 7 days after surgery and used as a measure of muscle loss. An open biopsy of the rectus femoris was taken under general anaesthetic by the surgical team prior to surgery. Demographic data and data associated with the procedure are given in *Table* 2. Rectus femoris rather than vastus lateralis biopsies were used in this study to enable direct comparison with the ultrasound data.

**Table 2:** Demographic characteristics of the cardiac surgery cohort

	Non‐wasting patients (*n* = 19)	Wasting patients (*n* = 21)	*P* value
Age (year)	58.7 ± 15.4	68.1 ± 14.9	0.04
Sex (M/F)	16/3	14/7	
BMI (kg/m^2^)	27.1 ± 3.6	27.5 ± 6.9	NS
EuroSCORE 2	2.0 (1.3–3.5)	3.0 (1.4–9.6)	NS
Pre‐operative LVEF %	59.6 ± 9.1	56.0 ± 10.6	NS
SPPB	12 (12, 12)	10 (10–12)	0.001
Total bypass time (min)	143.0 ± 49.0	142.1 ± 55.7	NS
Total cross‐clamp time (min)	103.6 ± 35.4	95.7 ± 33.2	NS
ICU length of stay (days)	1.0 (1–2)	3.0 (2–7)	<0.001
Hospital length of stay (days)	8 (7–12)	12 (9–23)	0.046
Mechanical ventilation (h)	16 (13–24)	26 (19–99)	0.01
Vasopressor duration (h)	27 (15–48)	46 (22–186)	0.019
MVC day 0 (kg)	26.4 ± 6.7	24.1 ± 9	NS
RF_CSA_ day 0 (cm^2^)	6.7 ± 2.2	6.1 ± 1.6	NS
RF_CSA_ % loss by day 7	2.8 (−0.49, 5.85)	13.6 (11.4, 20.6)	<0.001

Data are presented as mean ± SD for normally distributed data and as median (interquartile range) for data that is not normally distributed.

BMI, body mass index; LVEF, left ventricular ejection fraction; MVC, maximum voluntary contraction; RF_CSA_, rectus femoris cross‐sectional area; SPPB, Short Physical Performance Battery.

##### Cell culture and transfection

LHCN‐M2 cells were maintained in skeletal muscle growth medium as described by Zhu *et al*.^26^ For miRNA transfection, cells were seeded at a density of 5 × 10^4^/mL seeding 6250 cells in a 96‐well plate and scaling for the appropriate growth area. After 24 h, the cells were transfected with *miR*Vana™ (ThermoFisher Scientific) mimics using lipofectamine according to the manufacturer's instructions.

##### Ago2 pull‐down

LHCN‐M2 cells were grown in 10 cm culture dishes and transfected with either miR‐422a mimic or scrambled control and allowed to grow for a further 2 days. The cells were washed twice with ice‐cold PBS then lysed with cell lysis buffer (CLB, Promega) supplemented with protease inhibitor cocktail (Sigma). The lysate was pre‐cleared with G‐sepharose beads for 2 h at 4°C. The lysate was divided into two and incubated with the beads [prepared by pre‐incubation in CLB supplemented with salmon sperm DNA (0.2 mg/mL) and BSA (1 mg/mL)] and anti‐Ago2 antibody (Millipore, Billerica, MA., USA) or anti‐IgG antibody, for control, overnight at 4°C under rotation with 1× CLB. The beads were washed in CLB, once, IP buffer [50 mM Tris (pH 7.4), 5 mM MgCl_2_, 300 mM NaCl, 0.05% NP40] four times and PBS, once. RNA was extracted from the beads using the trizol method and cDNA synthesized. Data for each cDNA were normalized to GAPDH in the same sample and analysed as fold enrichment (anti‐Ago2/control IgG) for miR‐422a compared with scrambled miRNA.

##### TGF‐β signalling assays

LHCN‐M2 cells were transfected with *mir*Vana™ mimic then 24 h later with luciferase reporter vectors before being treated for 2 h with TGF‐β as described in the Supporting Information. TGF‐β assays were performed as previously described.^27^

##### Western blotting

Western blotting was performed as previously described^28^ and detailed in the Supporting Information.

##### Assessment of miRNA levels

RNA was extracted from muscle and blood using the trizol method as previously described.^28^, ^29^ miRNAs were determined using probes purchased from Applied Biosystems as previously described^12^ and detailed in the supplement.

### Statistical analyses

MicroRNA data were log transformed to standardize the variance. Data were inspected to identify linear associations; then, correlation analysis was performed using Pearson correlations (Aabel, Gigawiz). Differences between groups were calculated by Student's *t*‐test for normally distributed data and by Mann–Whitney *U* test for non‐parametric data (Aabel, Gigawiz).

## Results

### Targets of miR‐422a

Analysis of miRNA expression profiles in paired plasma and quadriceps samples from a screen cohort of 16 patients with COPD and 7 controls identified plasma miR‐422a as being both negatively associated with muscle strength and highly expressed in muscle (Supporting Information).

**Figure 1:**
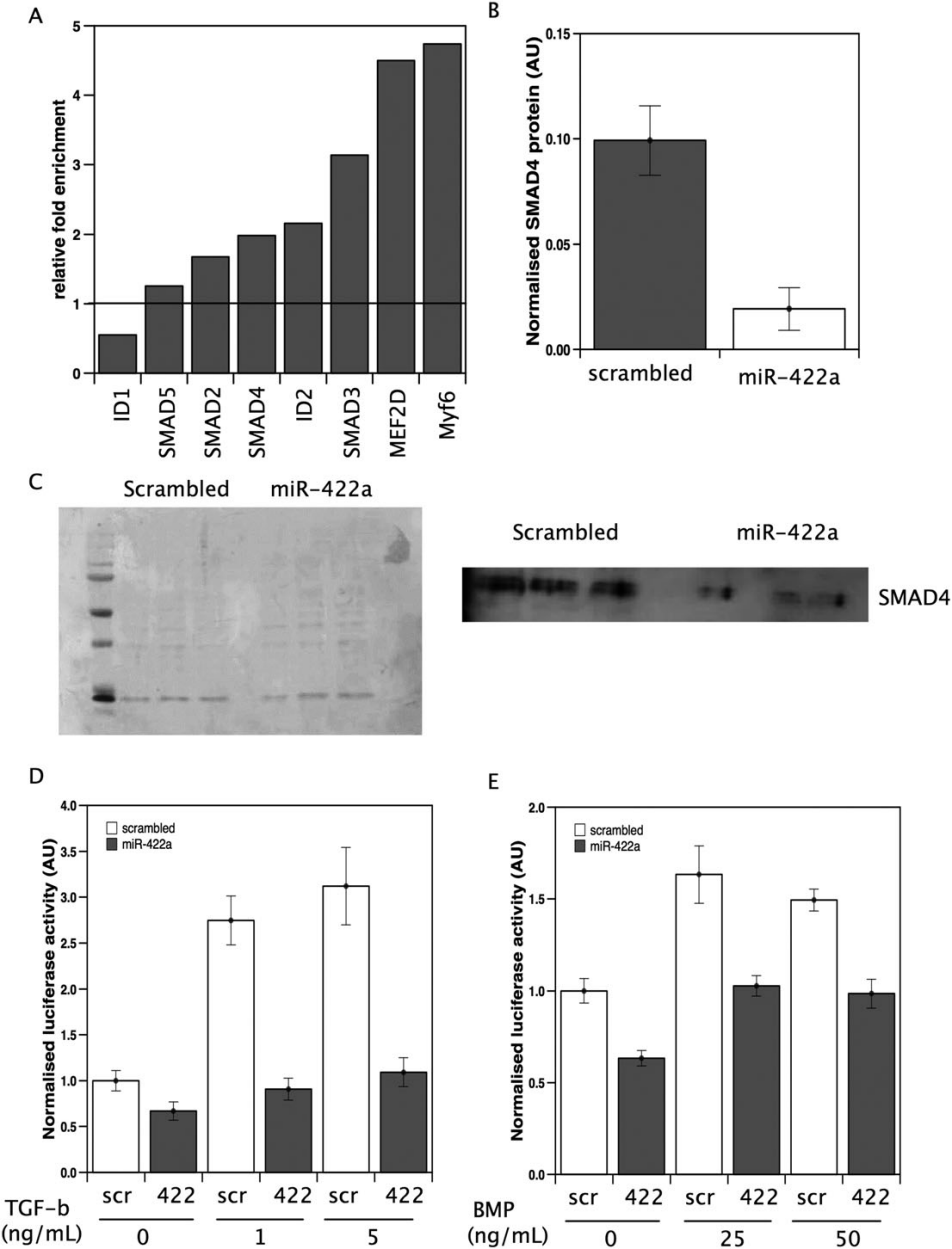
miR‐422a targets SMAD4 expression in muscle cells. (A) mRNAs for predicted miR‐422a targets were quantified in RNA co‐immunoprecipitated with anti‐Ago2 from cells transfected with miR‐422a or scrambled control as described in the Methods. Fold enrichment of each target was determined by normalizing the signals to a control gene within the sample and this value for miR‐422a‐transfected cells to scrambled‐transfected cells. The data represent average fold enrichment from two independent experiments. (B and C) LHCN‐M2 cells were transfected with miR‐422a mimic or scrambled control. Forty‐eight hours later, the cells were lysed and protein extracted. Western blots were quantified, and data normalised to total protein (Ponceau S stain, C; left‐hand panel). miR‐422a caused a marked reduction in SMAD4 expression. Data are taken from three independent transfections. LHCN‐M2 cells were transfected with miR‐422a mimic or scrambled control followed by the appropriate reporter constructs as described in Methods. Cells were treated with TGF‐β (D) or BMP (E) at the stated concentrations, and luciferase activity was measured 2 h later. TGF‐β increased luciferase at both 1 and 5 ng/mL. miR‐422a inhibited the increase in luciferase activity at both doses (*P* = <0.001 in both cases). BMP increased luciferase at both 25 and 50 ng/mL. miR‐422a suppressed basal and ligand‐stimulated luciferase activity. Data presented are from three independent experiments performed in hextuplicate.

A bio‐informatic analysis using miRwalk^21^ was performed to identify targets of miR‐422a likely to contribute to muscle phenotype. This analysis showed that miR‐422a targeted a large number of transcription factors including MEF‐2D, and Myf‐6. Interestingly, amongst these transcriptional regulators were SMAD3 and SMAD4, and we have previously shown that miR‐542‐5p, a miRNA that promotes SMAD2/3 phosphorylation^19^ and activation of SMAD signalling, is positively associated with muscle loss. We, therefore, determined whether SMAD4 was a target of miR‐422a in skeletal muscle cells using Ago2 pull‐down to isolate mRNAs bound to the RNA‐induced silencing complex in miR‐422a‐transfected cells. This analysis showed that SMAD4 was enriched in RNA pulled down using an Ago2 antibody from skeletal muscle cells transfected with the miRNA compared with RNA pulled down from cells transfected with a scrambled control miRNA (*Figure* 1A).

Transfection of LHCN‐M2 cells with miR‐422a did not reduce mRNA expression of SMAD4 compared with control transfection, but there was a marked reduction in the expression of SMAD4 protein as determined by western blotting (*Figure* 1B) which would result in inhibition of both TGF‐β and BMP signalling in cells. Transfection of LHCN‐M2 cells with miR‐422a did not inhibit basal luciferase activity driven from a CAGA_12_ promoter known to respond to TGF‐β compared with transfection with a scrambled control. In the presence of 1 and 5 ng/mL of TGF‐β, there was a 2.7 and 3.1 (*P* < 0.001 in both cases) fold increase in luciferase activity in cells transfected with control mimic, as we expected. However, in miR‐422a‐transfected cells, the presence of TGF‐β did not increase luciferase expression, indicating that miR‐422 ablates ligand‐induced TGF‐β signalling (*Figure* 1C).

The effect of miR‐422a on BMP signalling was determined by using a promoter containing a BMP‐response element driving luciferase. Luciferase activity from this promoter was lower under basal conditions in cells transfected with miR‐422a than in cells transfected with the control (*P* < 0.001). In the presence of 25 and 50 ng/mL BMP4, luciferase activity was increased 1.6 and 1.5‐fold in cells transfected with the scrambled control, again as expected. In miR‐422‐transfected cells, there was an increase in luciferase activity in the presence of BMP4 at both 25 and 50 ng/mL compared with cells transfected with miR‐422a in the absence of BMP4, but under all conditions, luciferase activity was lower than in cells transfected with scrambled oligonucleotide (*Figure* 1D). These data show that the expression of miR‐422a can reduce the expression of SMAD4 and suppresses ligand‐induced TGF‐β and basal and ligand‐induced BMP signalling.

### miR‐422a in muscle

Suppression of SMAD4 expression and of TGF‐β activity would be likely to inhibit muscle wasting in the presence of a myostatin signal and thereby preserve strength. We therefore determined the associations of miR‐422a expression with muscle mass and strength in patients with COPD, a condition where increased myostatin has been demonstrated^16^ and shown to be negatively associated with strength.^30^

**Figure 2:**
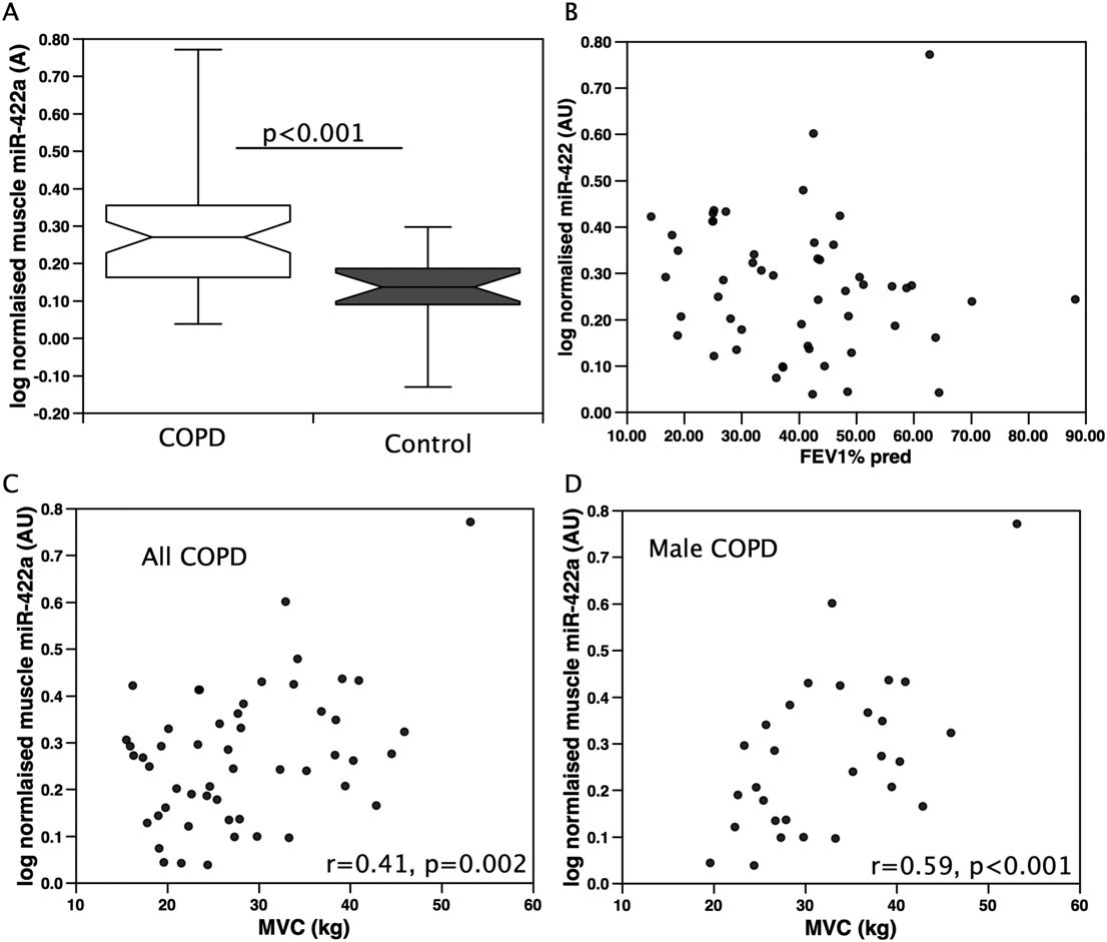
miR‐422a is positively associated with quadriceps strength in patients with COPD. miR‐422a was quantified in the quadriceps muscle of patients with COPD or age‐matched controls. miR‐422a was increased in patients with COPD compared with controls (A) but did not associate with disease severity FEV_1_ (B). miR‐422a was positively associated with strength in all patients with COPD (C), and this was stronger when males were considered alone (D).

Quadriceps miR‐422a expression was positively associated with muscle strength in patients (MVC%; *r* = 0.29, *P* = 0.039, MVC; *r* = 0.411, *P* = 0.002, *n* = 52, *Figure* 2). This correlation was stronger when considering males alone (MVC%; *r* = 0.40, *P* = 0.03, MVC; *r* = 0.59, *P* < 0.001, *n* = 29, *Figure* 2), and there was no association when considering the females alone. There was also no association in the small group of controls. Nine of the male samples were included in both the screen and validation cohorts, although the cDNA used in each case was different. Removal of these samples from the analysis did not remove the significance of association of miR422a with MVC% or MVC (*r* = 0.48, *P* = 0.034 and *r* = 0.64, *P* = 0.002). In the patients with COPD, there was no association of the miRNA with physical performance as assessed by 6 min walk distance expressed as % predicted. Contrary to our predictions, miR‐422a was significantly higher in the patients than the controls in the cohort as a whole, as well as in both sexes independently (*P* < 0.001 for the whole cohort, *Figure* 2, *P* = 0.020 for males and *P* = 0.008 for females, Mann–Whitney). Consistent with our starting observations, the miRNA did not associate with the patients' lung function measured as FEV_1_%, TLCO% or RV/TLC. The normalization data did not correlate with muscle strength or lung function (Supporting Information).

**Figure 3:**
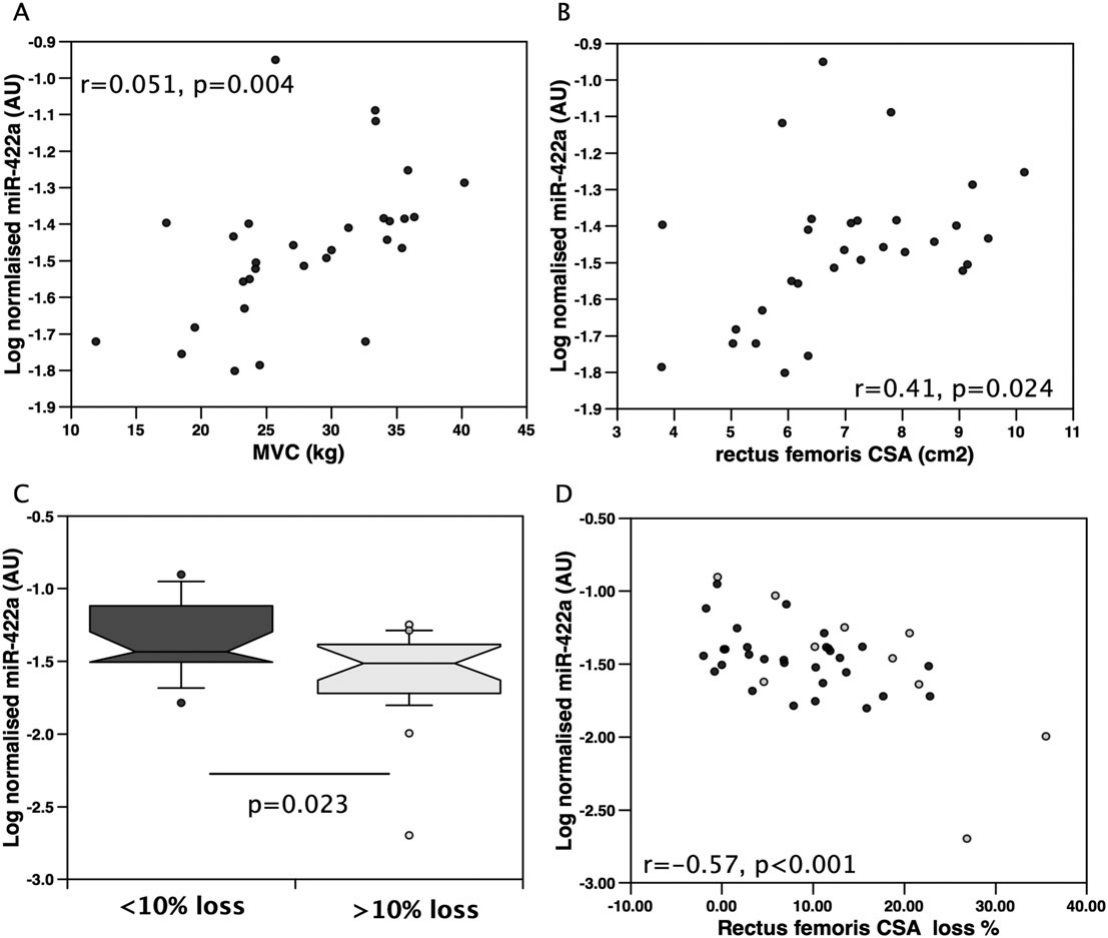
miR‐422a is positively associated with quadriceps strength and negatively associated with muscle loss in patients undergoing cardiac surgery. miR‐422a expression was determined in quadriceps samples from patients undergoing cardiac surgery. miR‐422a expression was positively associated with strength in men (*n* = 30, A) and with rectus femoris cross‐sectional area (RF_CSA_) in men (*n* = 30, B). (C) In all patients, miR‐422a expression was lower in those who lost more than 10% RF_CSA_ in 7 days following surgery (*n* = 19 non‐wasting and 21 wasting patients). (D) In all patients, there was a negative association of miR‐422a expression and muscle loss over 7 days. Black circles represent male patients, and grey circles represent female patients.

To determine whether there was a similar positive association of miR‐422a with muscle mass in patients with another chronic disease, we determined the expression of miR‐422a in patients with cardiac disease requiring aortic surgery. Comparison of the pre‐surgery expression of miR‐422a with physiological parameters in these patients showed that miR‐422a was positively associated with both strength and RF_CSA_ (*r* = 0.37, *P* = 0.019 and *r* = 0.33, *P* = 0.038, respectively, *Figure* 3), and again, these associations were stronger when males were considered alone (*r* = 0.51, *P* = 0.004, and *r* = 0.41, *P* = 0.024, respectively, *Figure* 3). Similarly, levels of miR‐422a expression did not show any association with measures of cardiac function in these patients.

Fifty per cent of these patients lose more than 10% of the RF_CSA_ in the 7 days following surgery. Pre‐operative levels of miR‐422a were lower in those who lost more than 10% of their RF_CSA_ than in those who did not (*P* = 0.023, *Figure* 3). Furthermore, pre‐operative expression of miR‐422a was inversely proportional to the RF_CSA_ loss over 7 days (*r* = −0.57, *P* < 0.001 for all patients, *Figure* 3, and *r* = −0.45, *P* = 0.012 for men only). Again, there was no association of the normalization data with muscle size or muscle loss in these patients (Supporting Information)

Due to a lack of appropriate samples, we were unable to determine the protein expression of SMAD4. However, as suitable RNA was available from 31 patients, we determined the expression of SMAD4 mRNA in the available samples. This analysis did not identify a difference in SMAD4 mRNA between those patients who lost muscle and those who did not, and there was no association in SMAD4 with muscle loss implying that any effect of the miRNA on SMAD4 *in vivo* would be at the translational level. Consistent with the lack of effect of miR‐422a on SMAD4 RNA levels, there was also no association of SMAD4 mRNA with miR‐422a.

## Discussion

Our data identify miR‐422a as a miRNA that is positively associated with muscle mass and strength in chronic disease. In response to a significant catabolic event (in this study, the stress of aortic surgery), individuals with high levels of miR‐422a lost less muscle than those with low levels of this miRNA. The *in vitro* observations suggest that miR‐422a confers resistance to catabolism by reducing the activity of the TGF‐β signalling system in the muscle, which is an effector of known mediators of muscle wasting like myostatin.

Activation of the SMAD signalling system can either promote muscle atrophy or hypertrophy dependent on the SMAD pathway activated.^31^, ^32^ Activation of SMAD2/3 by myostatin promotes muscle wasting, whereas activation of SMAD1/5 by BMPs favours myoblast proliferation and hypertrophy. Myostatin is increased in a number of chronic disease including COPD and cardiac diseases where muscle loss occurs. For example, myostatin expression is elevated in the muscle of patients with COPD^16^ and shows a weak inverse association with muscle strength.^1^ In some studies of heart failure, serum myostatin levels were increased in patients with heart failure,^33^, ^34^ whereas others failed to find such an increase.^35^ Consistent with SMAD2/3 signalling promoting muscle wasting, anti‐myostatin and anti‐activin IIB receptor antibodies reduced muscle loss in mouse models of muscle wasting,^36^, ^37^ and a subset of these agents have entered clinical trials (www.clinicaltrials.gov reference NCT01321320). Our data suggest that miR‐422a is an endogenous suppressor of the myostatin system in primates that regulates the expression of myostatin signalling pathway components and that this mechanism accounts for some of the variation in the muscle wasting response of individuals to a catabolic signal. Quantifying miR‐422a and/or SMAD4 protein may therefore help to identify individuals in whom anti‐myostatin treatments are likely to be most successful.

In addition to ligand‐dependent activation of SMAD signalling, we have recently shown a novel mode of activation of SMAD signalling by the microRNA miR‐542‐5p. Our data show that transfection of this miRNA into muscle cells causes SMAD2/3 phosphorylation and activation of the SMAD signalling system in the absence of exogenous ligand.^19^ Quadriceps expression of miR‐542‐5p is positively associated with disease severity in both patients with COPD in whom it is inversely proportional to lung function (measured as TLCO% predicted and FEV_1_% predicted) and in patients with reduced cardiac function, in whom it is inversely proportional to left ventricular ejection fraction. The ability of miR‐422a to suppress SMAD signalling suggests that patients with higher expression of miR‐422a would be relatively resistant to the effects of myostatin and to elevated miR‐542‐5p. This resistance would account for their maintenance of strength and muscle mass in both chronic and acute muscle wasting scenarios.

BMP signalling is generally considered to be pro‐hypertrophic, for example, BMP‐14 promoted hypertrophy and prevented excessive muscle loss in mice, signalling through SMAD1/5/8 and SMAD4.^31^ Consistent with this suggestion, mice that lack SMAD4 in their skeletal muscle are prone to muscle loss in response to denervation and to starvation,^31^ an observation at odds with our positive association of miR‐422a with muscle strength, yet targeting SMAD4. There are several possible explanations: Firstly, given the relatively high expression of miR‐422a, the direct association of miR‐422a and muscle mass is most likely to be derived from miRNA expressed in myofibres rather than myoblasts; secondly, because the expression of miR‐422a is unlikely to suppress the expression of SMAD4 completely; and thirdly because SMAD4 is not the only miR‐422a target. The cell type within which miR‐422a is expressed and acts is important because a major effect of BMPs in the maintenance of muscle mass is the inhibition of myoblast differentiation, thereby increasing the number of myoblasts available for recruitment to the muscle.^38^ Once the myoblasts have proliferated, factors that inhibit BMP signalling (e.g. miR‐675 which targets SMAD1/5/8) cause the myoblasts to withdraw from the cell cycle and differentiate thereby completing the regeneration process. Consequently, the effect of modifying the strength of BMP signalling on muscle mass is difficult to predict as both BMPs and inhibitors of BMPs are required to complete muscle regeneration. In the absence of sufficient myoblasts (caused by a weak BMP signal) or a failure of those myoblasts to fuse with the myofibres (caused by a failure to stop BMP signalling), myofibre regeneration will be impaired. The importance of balancing these processes can be seen by comparing the effects of miR‐675 in mice lacking H19, the non‐coding RNA that makes this miRNA^38^ with the effect of miR‐675 on myoblast proliferation *in vitro* and muscle mass in patients with COPD. In mice that lack H19, muscle regeneration is impaired and can be restored by injecting miR‐675 after the myoblasts have proliferated. Conversely, miR‐675 inhibits myoblast proliferation *in vitro*,^39^ and quadriceps expression of miR‐675 is inversely associated with muscle mass in chronic disease.^12^ Finally, the associations of miR‐422a with muscle mass and strength will be the net result of the effects of this miRNA on myofibre phenotype through multiple targets. For example, miR‐422a expression will also suppress p53 activity as it targets the co‐activator MLH‐1,^40^ and previous studies have shown that fibres lacking p53 are resistant to atrophy.^41^ As a result in the context of chronic disease and following surgery, partially inhibiting the effects of myostatin may be of greater effect than partially inhibiting the effects of BMPs.

The reason for miR‐422a associating with strength and muscle mass in men but not in women is not clear, but the most obvious route for such a difference between sexes is through androgen signalling. Previous studies have shown crosstalk between the TGF‐β signalling system and androgen receptor function in prostate cells raising the possibility of a contribution of androgens to our observation. Perhaps most relevant is the observation that both SMAD3 and SMAD4 can directly interact with the androgen receptor in the absence of added TGF‐β. SMAD3 alone acts as a co‐activator of the androgen receptor, whereas in combination with SMAD4, it acts as a repressor. If the same responses occur in muscle cells, it is possible that by titrating down SMAD4, miR‐422a increases the relative activity of the androgen receptor.^42^

We have previously observed that miR‐519a is also positively associated with FFMI in male patients with COPD but not in female patients with COPD.^12^ As the miRNAs from the C19MC are regulators of pluripotency, it is also possible that the relative contribution of satellite cell proliferation and recruitment to muscle mass differs in men and women. As such, factors that modify satellite cell number or myoblast proliferation and differentiation may also have differential effects on the maintenance of muscle mass in men and women. Consistent with differences in satellite cell recruitment between males and females, two studies have reported greater numbers of centralized nuclei in biopsies from men compared with women.^43^, ^44^

#### Critique of the study

Our data show that the expression of miR‐422a is positively associated with muscle strength in patients with chronic diseases but are cross‐sectional in nature. They cannot therefore demonstrate causality. Furthermore, given the restriction of miR‐422a to primates, it is not easy for us to study the role of this miRNA directly in an animal model. However, our longitudinal study shows that low levels of miR‐422a are predictive of muscle loss in humans undergoing surgery, and our *in vitro* studies provide plausible mechanisms by which this miRNA may contribute to the maintenance of muscle mass. A major limitation of the study is our inability to quantify SMAD4 protein in muscle biopsy samples. It is therefore not possible for us to confirm that the suppression of this protein is the major cause of the associations of miR‐422a that we observed in this study.

## Conclusions

Our data identify miR‐422a as a miRNA that is positively associated with strength in two patient cohorts and with muscle wasting in a longitudinal study. We also identify miR‐422a as a suppressor of TGF‐β signalling by reducing the expression of SMAD4 providing a potential mechanism by which this miRNA contributes to the maintenance of muscle mass.

## Conflict of interest

M.I.P. reports personal fees from GSK and grants and personal fees from Novartis, outside the submitted work; M.G. reports grants, personal fees and non‐financial support from GSK, personal fees from BI, personal fees from Silence Therapeutics and personal fees from Cell Catapult, outside the submitted work; all other authors have no conflict of interest.

## Contributions

The overall study was designed by P.K. with contributions from M.G. and M.I.P. Patients were recruited, and samples were collected by R.P., U.R. and S.A.N. The laboratory studies were performed by M.S., M.C., R.P., A.D. and J.Y.L. P.K. wrote the first draft of the paper, and all authors provided critical appraisal and input into the manuscript.

## Supporting information


**Figure S1.** Circulating miR‐422a is inversely associated with strength and activity in patients with chronic obstructive pulmonary disease.

**Figure S2.** RNU48 levels were not associated with muscle strength or performance in patients with chronic obstructive pulmonary disease.

**Figure S3.** Normalizer values were not associated with strength in male patients with chronic obstructive pulmonary disease.

**Figure S4.** Normalizer values were not associated with strength or muscle loss following aortic surgery.

**Table S1.** Patient demographics for the screen cohort (all male)
**Table S2.** miRNAs different between patients with a low fat free mass index and those with a normal fat free mass index
**Table S3.** Patient demographics of plasma cohort
