# Supplementary figures and images for: miR‐422a suppresses SMAD4 protein expression and promotes resistance to muscle loss
Source: J Cachexia Sarcopenia Muscle. 2017 Oct 6;9(1):119–28. doi: 10.1002/jcsm.12236 (PMC5803610; doi:10.1002/jcsm.12236)

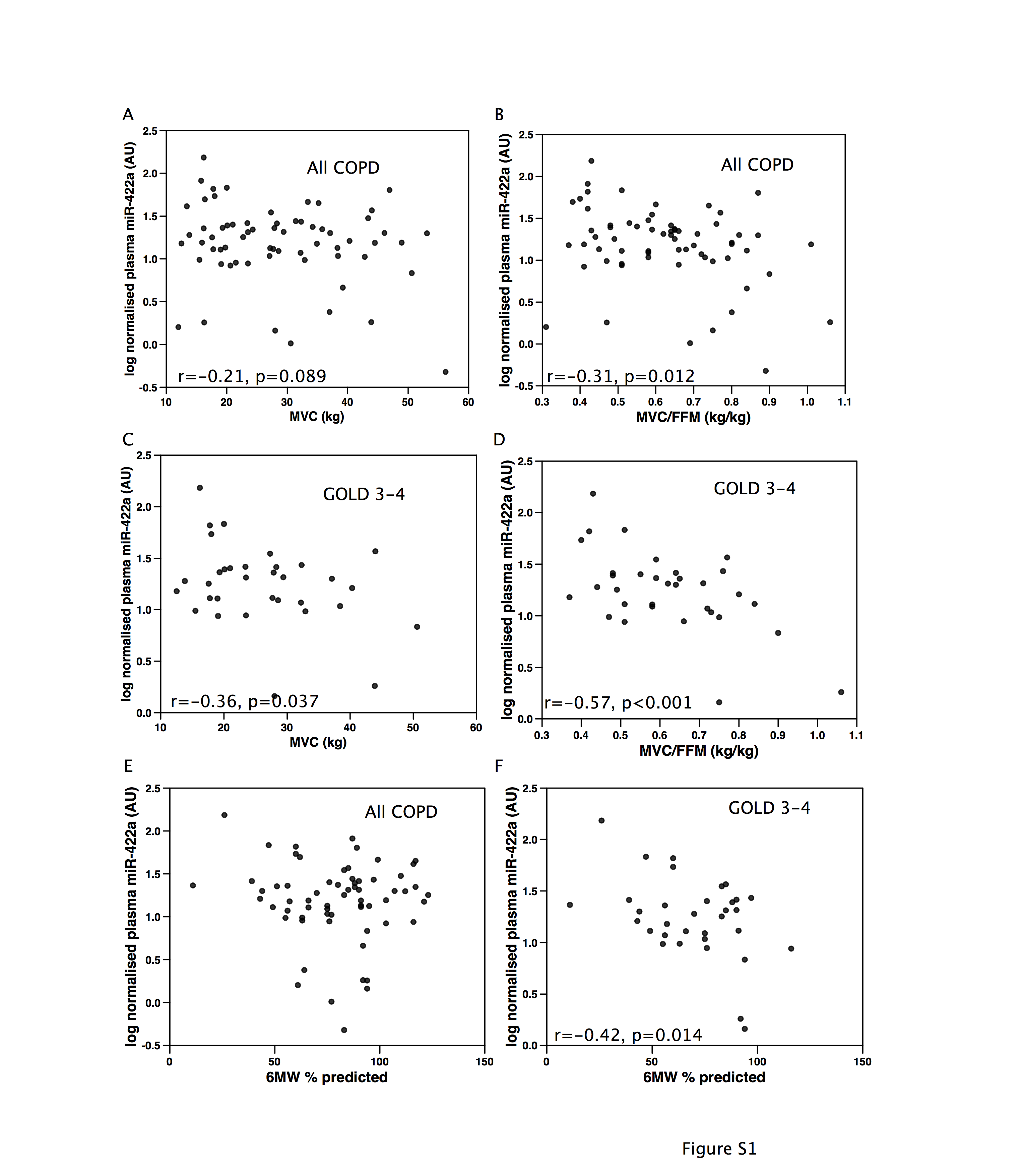

Supplement: Supplementary file 1 — Figure S1. Circulating miR‐422a is inversely associated with strength and activity in patients with chronic obstructive pulmonary disease. [file JCSM-9-119-s001.tif]

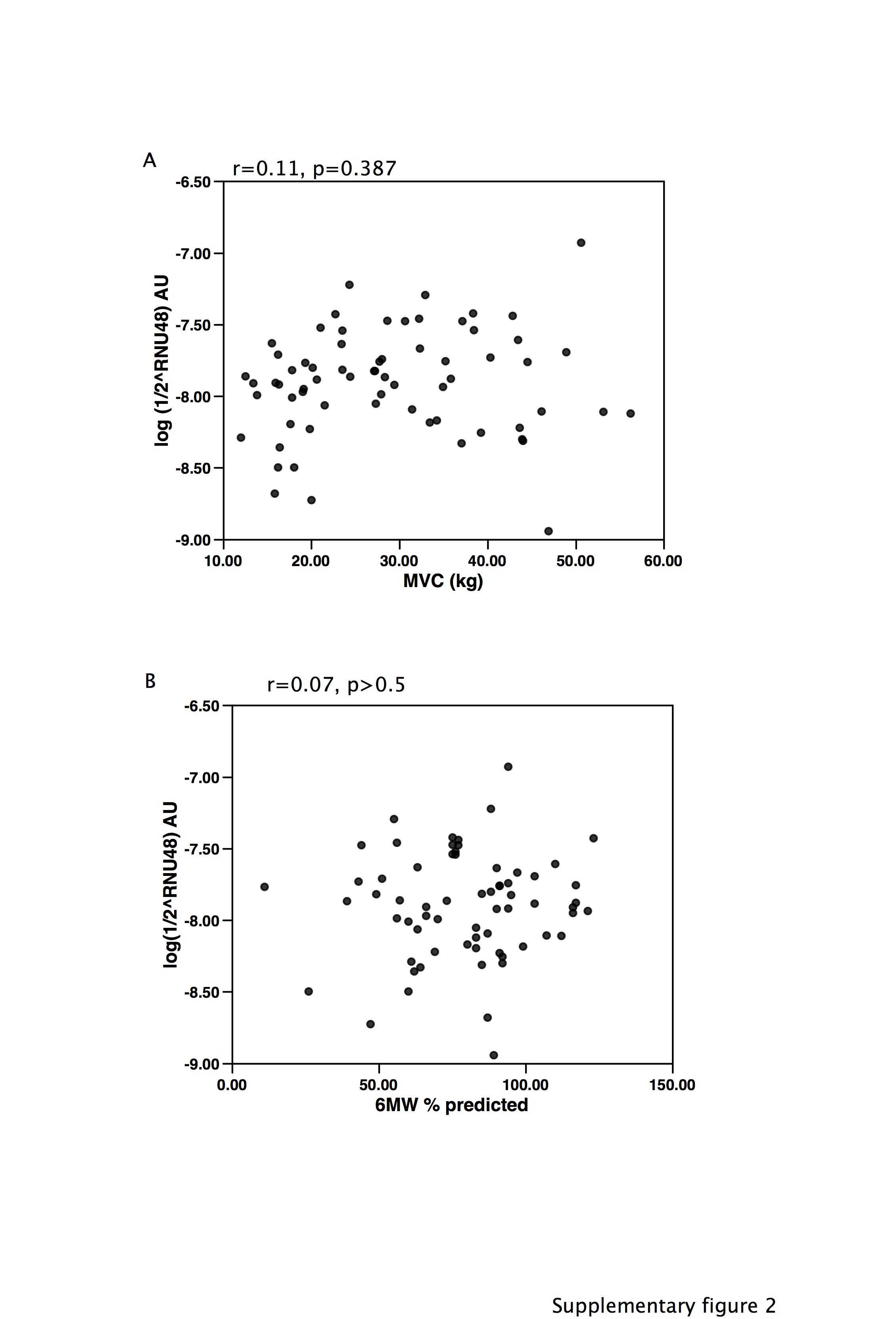

Supplement: Supplementary file 2 — Figure S2. RNU48 levels were not associated with muscle strength or performance in patients with chronic obstructive pulmonary disease. [file JCSM-9-119-s002.tif]

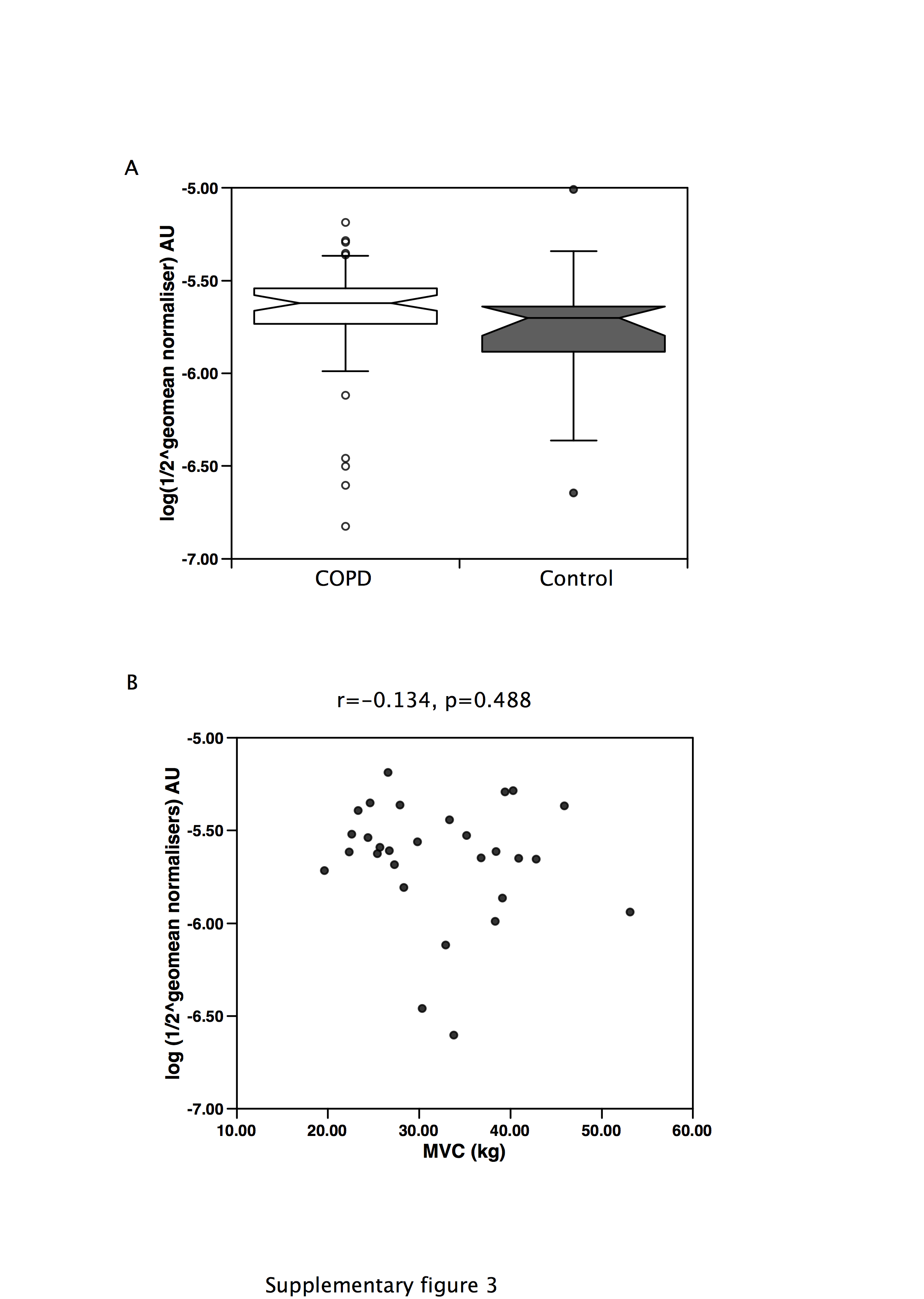

Supplement: Supplementary file 3 — Figure S3. Normalizer values were not associated with strength in male patients with chronic obstructive pulmonary disease. [file JCSM-9-119-s003.tif]

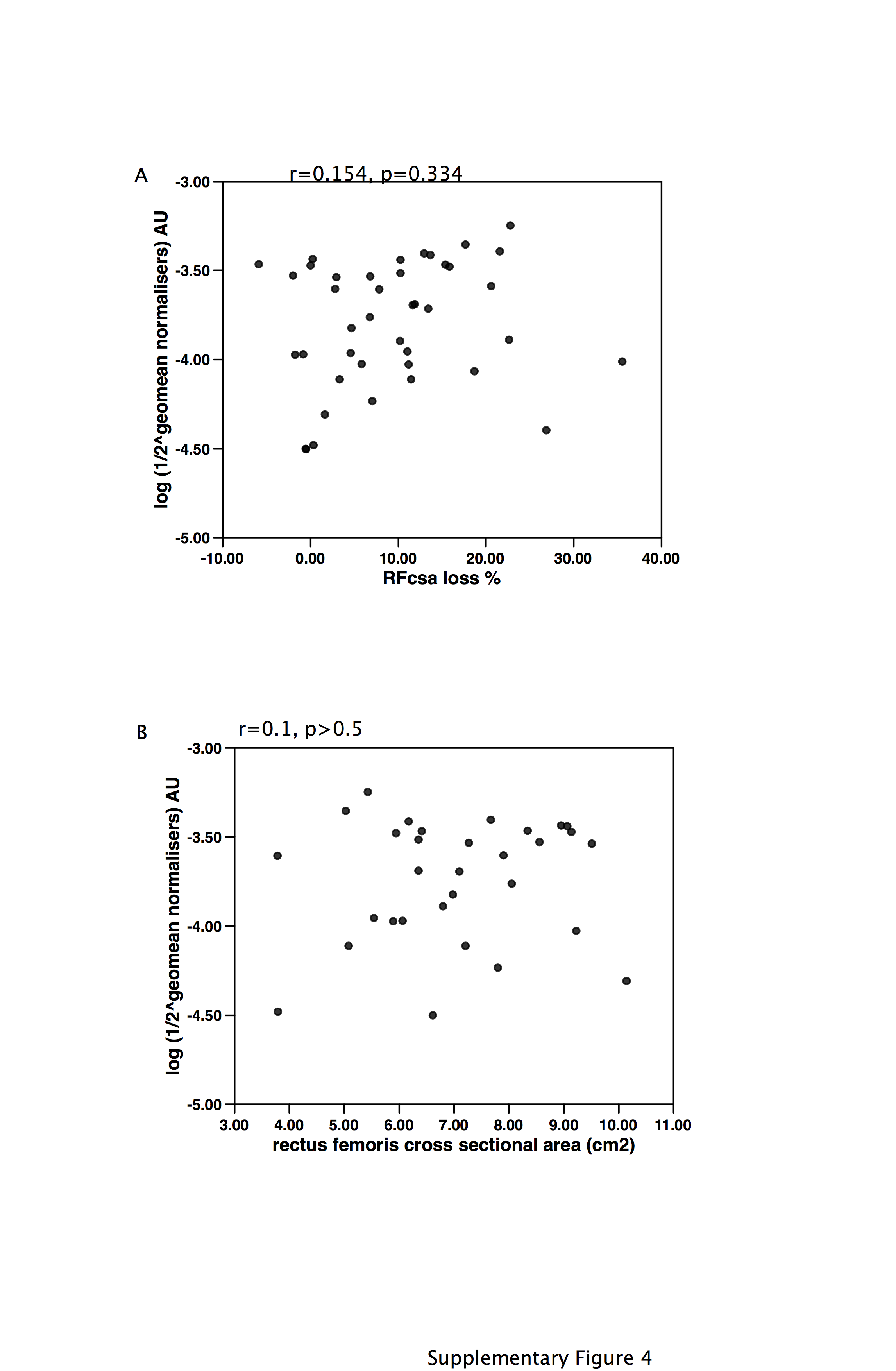

Supplement: Supplementary file 4 — Figure S4. Normalizer values were not associated with strength or muscle loss following aortic surgery. [file JCSM-9-119-s004.tif]
