# Supplementary material for: miR‐422a suppresses SMAD4 protein expression and promotes resistance to muscle loss
Source: J Cachexia Sarcopenia Muscle. 2017 Oct 6;9(1):119–28. doi: 10.1002/jcsm.12236 (PMC5803610; doi:10.1002/jcsm.12236)
Supplement: Supplementary file 5 — Table S1. Patient demographics for the screen cohort (all male) Table S2. miRNAs different between patients with a low fat free mass index and those with a normal fat free mass index Table S3. Patient demographics of plasma cohort [file JCSM-9-119-s005.docx]

**Supplementary information**

**Supplementary methods**

**TGFβ assays:** 24h after transfection with the *mir*Vana^TM^ mimic, cells were transfected with the luciferase reporter vectors according to the manufacturer’s instructions. In each well of a 96-well plate 0.094μg of vector CAGA_12_-luc and 0.031μg of pRL-TK were used. 24h later cells were washed twice with serum free DMEM before the addition of TGF-β. After 2h the cells were harvested and luciferase activity measured using the DualGlo luciferase assay (Promega). Firefly luciferase was normalised to Renila luciferase and the experiments were performed in sextuplet and repeated three times.

**Western Blotting**

LHCN-M2 cells were harvested and lysed 48h after transfection with the *mir*Vana^TM^ mimic. 25μg of protein was analysed by SDS-PAGE on 10% acrylamide gels then transferred to PVDF membranes. Membranes were incubated with 5% milk in tris buffered saline supplemented with 0.1% Tween-20 (TBS-T) for 1h then overnight at 4°C with anti-SMAD4 (Santa Cruz H-552) in diluted 1:500 in TBS-T with 5% milk. After washing in TBS-T (3 times for 5 min per wash) the blot was incubated with HRP conjugated anti-rabbit IgG (1:1000) for 1 day before washing (3 times for 5 min per wash) and visualisation using the ECL plus detection kit (GE Healthcare). Blots were stained with Ponceau S solution to confirm even loading.

***Blood sampling:*** Venous blood was collected into EDTA tubes and centrifuged at 1,500 x g (3500 rpm) for 10 minutes, within 2 hours of collection. Plasma was separated from the cell pellet and stored frozen at -80°C until used.

**RNA analysis**

***RNA extraction***

RNA including small RNAs were extracted from plasma and cells using Qiazol or trizol respectively and as previously described [^1^](#_ENREF_1) [^2^](#_ENREF_2)

***Assessment of mRNA and miRNA levels*:** Messenger RNA was quantified by quantitative real time PCR (qRTPCR) as described previously [^3^](#_ENREF_3). For the analysis of microRNA expression, the RNA was reverse transcribed using MultiScribe™ Reverse Transcriptase with, Megaplex™ RT Primers (human pools A, Version 3.0, Applied Biosystems) according to the manufacturer’s instructions. The reactions were incubated at 85°C for 5min to inactivate the enzyme and the cDNA stored at -80°C . The cDNAs were pre-amplified using Megaplex™ PreAmp Primers (Applied Biosystems) for 12 cycles of 95°C for 15 sec and 60°C for 4 min. The reaction was terminated by heating to 99.9°C for 10 min and pre-amplified cDNA diluted by addition of 75μl of 0.1× TE buffer pH 8.0 (Qiagen) and stored at -80°C.

***TaqMan® Array MicroRNA Cards*:** The amplified cDNAs were quantified using the Human TaqMan® Array MicroRNA Card A (V.3.0 Applied Biosystems) using an ABI 7900HT thermocycler, according to the manufacturer’s instructions, using the 384 well TaqMan® Low Density Array default thermal-cycling conditions defined by SDS 2.4 software. The data from all cards were calibrated to a single control sample with one detection threshold set across all samples and assays using RQ manager software. The resultant Ct values were then exported and normalised to the geometric mean of U6, RNU44 and RNU48 for the muscle and U6 and RNU 48 for plasma, using the ΔΔ Ct method. As variance tended to increase with miRNA intensity the data were standardised by a log transformation.

***Quantification of single microRNAs using TaqMan® probes*:** Individual microRNAs were quantified using custom designed primers and probes for each test gene (Applied Biosystems). RNA was reverse transcribed and cDNA preamplified as described above according to the manufacturer’s instructions. Each reaction was performed in duplicate and the average Ct value normalised to the corresponding geometric mean of U6 and RNU48 for the muscle and to RNU48 for plasma, using the ΔΔ Ct method.

**Supplementary Results**

**Screen of miRNAs in circulation**

To identify miRNAs associated with muscle mass in the circulation of male patients with COPD, RNA was isolated from the plasma of 16 patients with GOLD3/4 COPD. 8 of the patients had a low FFMI (LFFMI) whereas the other 8 patients had an FFMI within the normal range (NFFMI) using the LifeTechnologies A card. One card failed (from the NFFMI group) and this patient was therefore excluded. The demographics for the groups are given in Table S1 but all groups were matched for age and the COPD groups only differed with regards to their muscle mass (by design) and consequently their weight and BMI.

Comparison of circulating levels of miRNAs identified 12 miRNAs that were different between patients with a low FFMI and those with a normal FFMI at p<0.05 (Table S2). This low p value cut-off was necessary as only one miRNA achieved a significance of <0.01. However, several of the miRNAs that were significant to p<0.05 shared significant features suggesting that the differences were not purely due to chance. For example, 9 were derived from imprinted regions of the genome; 2 from a paternally imprinted cluster on chromosome 19 (C19MC) that we have previously shown are suppressed in the muscle of men with severe COPD and a LFFMI compared to those with severe COPD and a normal FFMI and 6 from a maternally imprinted locus on chromosome 14. The association of these miRNAs as markers of muscle mass and function will be discussed elsewhere. From the non-imprinted miRNAs we selected miR-422a for further analysis as it was readily detectable in muscle raising the possibility that the miRNA in plasma was derived from the muscle pool.

To determine whether plasma miR-422a was associated with muscle mass or function in COPD we analysed its levels in a larger group of patients of both sexes and of all GOLD stages as well as controls (Table S3). In these samples miR-422a was not associated with FFMI in the cohort as a whole or in any sub group divided by sex or disease severity. miR-422a was also not associated with muscle strength as MVC in all of the patients (r=-0.21, p=0.089, n=66, Fig S1A) but was associated when MVC was normalised to FFM (r=-0.31, p=0.012, Fig. 1B). Both associations were also stronger when the cohort was restricted to the patients with the most severe disease (GOLD 3-4 r=-0.36, p=0.037, and r=-0.57, p<0.001 respectively, n=33, Fig S1C and D). Furthermore, in these more severe patients miR-422a was inversely correlated with physical performance measured as 6-minute walk distance predicted (6MW%, r=-0.42, p=0.014, n=33, Fig. S1E and F). However, there was no association of miR-422a with 6MW% when all patients were considered together.

**Analysis of the normalisation data for PCRs**

To determine whether the normalisation process could have driven any of the associations of mIR-422a with physiological factors, we determine analysed the level of the chosen normalisers against strength, muscle mass and muscle loss. To use the same value for the normaliser as is used in the 2^Delta-Ct^ calculation we used 1/2^Ct^ for each normaliser or the geometric mean of the appropriate normaliser values.

Plasma RNU48 was not associated with strength measured as MVC or MVC/ FFM in either the whole COPD cohort (p=0. 386 and p=0.179 respectively) or in the GOLD 3-4 patients alone (r=0.29 p=0.086 and r=0.27 p=0.115 respectively), nor was RNU48 associated with 6MW % predicted in either group (r=0.07, p>0.5 and r=0.15, p=0.395 respectively).

Neither RNU48 and U6 nor the geometric mean of was different between COPD patients and controls p=0.115 (shown for geomean). There was no association of the normalisers alone or as the geomean with MVC in either the whole COPD cohort (geomean r=0.09, p>0.5) or the COPD men alone (geomena r=-0.13, p=0.488). Similarly in the aortic surgery cohort there was no association of the normalisers or the geometric mean of the normalisers with muscle size (RF_CSA_) in the men (geomean r=0.1, p>0.5) or muscle loss (RF_CSA_ % loss) in the whole cohort (r=0.16, p=0.334).

Neither of the HPRT and RPLPO normalisers nor their geometric mean were associated with RF_CSA_ loss or muscle size (geomean r=-0.34, p=0.058, and r=-0.06, p>0.5).

**
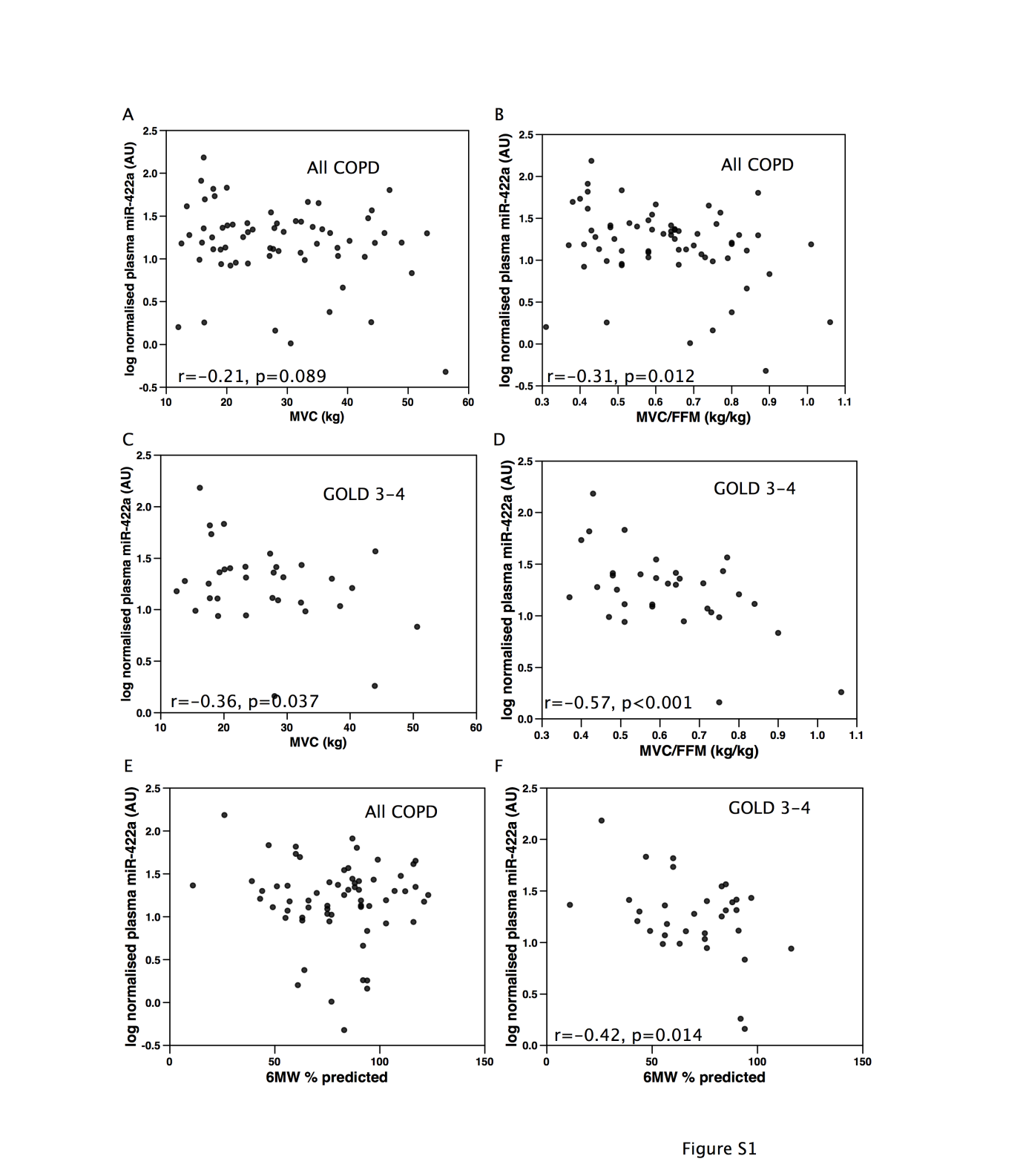
Figure S1 Circulating miR-422a is inversely associated with strength and activity in COPD patients**

miR-422a was quantified in the plasma of a cohort of 66 COPD patients. (A and B) Levels of the mRNA were not associated with strength (maximum voluntary contraction) when all patients were considered but were negatively associated with MVC normalised for fat free mass (FFM). (C and D) miR-422a was negatively associated with both MVC and MVC normalised for FFM in patients with severe COPD. miR-422a levels were not associated with physical performance (6-minute walk distance) in all patients (C) but were negatively associated in patients with severe COPD (D).

**Figure S2 RNU48 levels were not associated with muscle strength or performance in COPD patients**

RNU48 was quantified in the plasma of patients as a normaliser for extraction and RT efficiency for the analysis of miR-422a. To confirm that associations described in Figure S1 were a consequence of miR-422a and not driven by anomalies in the RNU48 levels, values used for RNU48 were compared to the physiological parameters shown to associate with normalised miR-422a. There was no association of RNU48 with (A) muscle strength or (B) predicted 6 minute walk distance.

**Figure S3 Normaliser values were not associated with strength in male COPD patients**

The geometric mean of RNU48 and U6 quantified in the muscle of patients was used as a normaliser for extraction and RT efficiency for the analysis of miR-422a. To confirm that associations described in Figure 2 were a consequence of miR-422a and not driven by anomalies in this value, the geometric mean of the normalisers was compared with the patients and controls and against strength. There was no difference in levels of the normalisers or the geomean of these values (A) between patients and controls, nor did the values correlate with strength in the whole group (not shown) or in men (B).

**Figure S4 Normaliser values were not associated with strength or muscle loss following aortic surgery**

The geometric mean of RNU48 and U6 quantified in the muscle of patients was used as a normaliser for extraction and RT efficiency for the analysis of miR-422a. To confirm that associations described in Figure 2 were a consequence of miR-422a and not driven by anomalies in this value, the values for each normaliser (not shown) and their geometric mean was compared with muscle loss following surgery (A) and with strength in men (B). No associations were identified by this analysis.

**Table S1: Patient demographics for the screen cohort (all male).**

|  | LFFMI (n=8) | NFFMI (n=7) | p |
| --- | --- | --- | --- |
| Age | 68 ± 6 | 64 ± 4 | NS |
| Smoking history (pack yr) | 59 ± 30 | 57 ± 26 | NS |
| Weight (kg) | 62 (55, 69) | 77 (69, 93) | 0.049 |
| BMI (kg/m^2^) | 21.4 (19.9, 22.7) | 24.9 (24.2, 26.2) | 0.021 |
| FFMI (kg/m^2^) | 14.5 (13.9 , 14.8) | 16.5 (16.4, 17.1) | <0.001 |
| FEV1% | 29.6 (27.3, 31.6) | 25.1 (18.9, 40.4) | NS |
| TLCO% | 30.5 ± 17.7 | 41.1 ± 15.9 | NS |
| 6MW% | 65 ± 24 | 72 ± 29 | NS |
| MVC (kg) | 24.1 (20.3, 32.6) | 30.3 (22.6, 38.4) | NS |
| MVC/FFM (kg) | 0.58 (0.53, 0.76) | 0.58 (0.49, 0.70) | NS |

BMI; body mass index, FFMI; fat free mass index, FEV_1_%; Forced Expiratory volume in 1 second (% of predicted value), TLCO; transfer capacity of the lung for CO (% of predicted value), 6MWD%; 6 minute walk distance (% of predicted value), MVC; maximum voluntary contraction. LFFMI; low fat free mass index (FFMI<16kg/m^2^), NFFMI; normal fat free mass index (FFMI <16kg/m^2^). Data are presented as mean ± SD for normally distributed data and as median (interquartile range) for data that is not normally distributed.

**Table S2: miRNAs different between patients with a low FFMI and those with a normal FFMI**

| miRNA | fold change NFFMI/LFFMI | p value |
| --- | --- | --- |
| miR-539 | 2.61 | 0.002 |
| miR-485-3p | 2.22 | 0.024 |
| miR-135b | 3.55 | 0.025 |
| miR-518e | 2.74 | 0.028 |
| miR-487b | 2.45 | 0.029 |
| miR-519d | 53.13 | 0.032 |
| miR-485-5p | 13.34 | 0.038 |
| miR-210 | 1.84 | 0.040 |
| miR-422a | 2.44 | 0.043 |
| miR-655 | 2.13 | 0.045 |
| miR-134 | 2.42 | 0.049 |
| miR-520g | 0.22 | 0.049 |

**Table S3 patient demographics of plasma cohort**

|  | COPD (n=65) | Control (n=15) | p |
| --- | --- | --- | --- |
| Age | 67 ± 9 | 66 ± 8 | NS |
| Male/Female | 31M, 34F | 5M, 10F |  |
| Smoking history (pack yr) | 45 (35,55) | 0 (0, 8) | <0.001 |
| Weight (kg) | 71 (59,83) | 65 (61, 76) | NS |
| BMI (kg/m^2^) | 24.8 (21.9, 29.1) | 24.8 (24.0, 26.2) | NS |
| FFMI (kg/m^2^) | 16 (14.4, 17.5) | 15.8 (15.2, 17.9) | NS |
| FEV1% | 49.4 ± 19.8 | 111.7 ± 12.7 | <0.001 |
| TLCO% | 45.6 (28.2, 59.3) | 86.7 (79.0, 95.3) | <0.001 |
| 6MW% | 79.6 ± 23.7 | 126.0 ± 10.7 | <0.001 |
| MVC (kg) | 27.7 (19.1, 37.0) | 33.5 (27.3, 43.6) | NS |
| MVC/FFM (kg) | 0.62 ± 0.17 | 0.74 ± 0.12 | 0.017 |

BMI; body mass index, FFMI; fat free mass index, FEV_1_%; Forced Expiratory volume in 1 second (% of predicted value), TLCO; transfer capacity of the lung for CO (% of predicted value), 6MWD%; 6 minute walk distance (% of predicted value), MVC; maximum voluntary contraction. Data are presented as mean ± SD for normally distributed data and as median (interquartile range) for data that is not normally distributed.

1. Lewis A, Riddoch-Contreras J, Natanek SA, Donaldson A, Man WD, Moxham J, et al. Downregulation of the serum response factor/miR-1 axis in the quadriceps of patients with COPD. *Thorax* 2012;67(1):26-34.

2. Donaldson A, Natanek SA, Lewis A, Man WD, Hopkinson NS, Polkey MI, et al. Increased skeletal muscle-specific microRNA in the blood of patients with COPD. *Thorax* 2013;68:1140-9.

3. Ellis PD, Smith CW, Kemp P. Regulated tissue-specific alternative splicing of enhanced green fluorescent protein transgenes conferred by alpha-tropomyosin regulatory elements in transgenic mice. *J Biol Chem* 2004;279(35):36660-9.
